# The Emerging Importance of Tumor Genomics in Operable Non-Small Cell Lung Cancer

**DOI:** 10.3390/cancers13153656

**Published:** 2021-07-21

**Authors:** Harry B. Lengel, James G. Connolly, Gregory D. Jones, Raul Caso, Jian Zhou, Francisco Sanchez-Vega, Brooke Mastrogiacomo, James M. Isbell, Bob T. Li, Yuan Liu, Natasha Rekhtman, David R. Jones

**Affiliations:** 1 Thoracic Surgery Service, Department of Surgery, Memorial Sloan Kettering Cancer Center, New York, NY 10065, USA; lengelh@mskcc.org (H.B.L.); jgc9012@nyp.org (J.G.C.); gdj9003@nyp.org (G.D.J.); Raul.Caso@medstar.net (R.C.); sanchezf@mskcc.org (F.S.-V.); mastrogb@mskcc.org (B.M.); isbellj@mskcc.org (J.M.I.); liuy1@mskcc.org (Y.L.); 2 Department of Surgery, Peking University, Beijing 100081, China; zhoujian@bjmu.edu.cn; 3 Department of Medicine, Memorial Sloan Kettering Cancer Center, New York, NY 10065, USA; lib1@mskcc.org; 4 Druckenmiller Center for Lung Cancer Research, Memorial Sloan Kettering Cancer Center, New York, NY 10065, USA; rekhtman@mskcc.org; 5 Department of Pathology, Memorial Sloan Kettering Cancer Center, New York, NY 10065, USA

**Keywords:** next-generation sequencing, surgery, lung cancer

## Abstract

**Simple Summary:**

Next-generation sequencing (NGS) has revolutionized care for patients with advanced and metastatic non-small cell lung cancer through the identification of specific oncogenic driver mutations and pairing with matched targeted therapies. The application of NGS technologies also has the potential to improve outcomes in patients with earlier-stage disease who undergo surgery as their first line of treatment. We review clinically relevant topics in this patient cohort, for whom NGS technologies have spearheaded our understanding of tumor heterogeneity, the underlying genomic features associated with lung adenocarcinoma histologic subtypes, the prediction of recurrence after surgery, the identification of minimal residual disease by circulating tumor DNA, the discernment of intrapulmonary metastases versus synchronous or metachronous disease, and the identification of patients with early-stage non-small cell lung cancer who are likely to benefit from induction or adjuvant therapies.

**Abstract:**

During the last two decades, next-generation sequencing (NGS) has played a key role in enhancing non-small cell lung cancer treatment paradigms through the application of “targeted therapy” in advanced and metastatic disease. The use of specific tyrosine kinase inhibitors in patients with oncogenic driver alterations, such as *EGFR*, *ALK*, *ROS1*, *BRAF* V600E, *MET*, and *NTRK* mutations, among others, has changed treatment approaches and improved outcomes in patients with late-stage disease. Although NGS technology has mostly been used in the setting of systemic therapy to identify targets, response to therapy, and mechanisms of resistance, it has multiple potential applications for patients with earlier-stage disease, as well. In this review, we discuss the emerging role of NGS technologies to better understand tumor biology in patients with non-small cell lung cancer who are undergoing surgery with curative intent. In this patient cohort, we examine tumor heterogeneity, the underlying tumor genomics associated with lung adenocarcinoma subtypes, the prediction of recurrence after complete surgical resection, the use of plasma circulating tumor DNA for detection of early cancers and monitoring for minimal residual disease, the differentiation of separate primaries from intrapulmonary metastases, and the use of NGS to guide induction and adjuvant therapies.

## 1. Introduction

The identification of specific oncogenic drivers through next-generation sequencing (NGS) and the development of matched targeted therapies have revolutionized cancer care and associated outcomes for patients with locoregionally advanced and metastatic non-small cell lung cancer (NSCLC). Clinical trials initiated in the mid-2000s in patients with stage IV *EGFR*-mutant lung adenocarcinoma (LUAD) treated with specific tyrosine kinase inhibitors (TKIs) offered the initial proof of concept for this “targeted therapy” approach [1,2,3]. During the last 15 years, additional NGS-identified oncogenic drivers, such as *ALK*, *ROS1*, *BRAF* V600E, *MET*, and *NTRK*, have been identified, as have additional first-, second-, and third-generation TKIs [4]. The subsequent clinical trials in stage IV NSCLC have demonstrated markedly improved patient outcomes with TKI approaches that target NGS-identified drivers, compared with platinum-based chemotherapy alone [5].

Despite NGS-driven advances in cancer care for locoregionally advanced and metastatic NSCLC, fewer efforts have been made to leverage tumor genomic profiling for earlier-stage NSCLC, where surgery plays a prominent diagnostic and therapeutic role. Heretofore, NGS-based diagnostics and therapeutics have primarily focused on identification of targets, response to therapy, and mechanisms of acquired TKI resistance. However, for stage I–IIIA NSCLC, there are other clinically relevant questions that the application of NGS technologies can help to answer. Examples of important areas relevant to early-stage NSCLC include (1) a better understanding of tumor heterogeneity, (2) the underlying genomic features associated with the tumor biology of LUAD histologic subtypes, (3) prediction of recurrence of disease after surgery, (4) identification of minimal residual disease (MRD), (5) discernment of intrapulmonary metastases (IPMs) from synchronous or metachronous disease, and (6) identification of patients with early-stage NSCLC who are likely to benefit from induction or adjuvant targeted therapies.

As care paradigms for advanced-stage lung cancer are rapidly changing, there is an expectation, and actual anticipation, that the use of similar genomic- and molecular-based approaches will improve outcomes among patients with earlier-stage disease. In this review, we discuss the emerging role of NGS-based technologies and their applications in early-stage NSCLC, with a focus on patients undergoing surgery with curative intent.

## 2. Tumor Heterogeneity and Evolution

It is increasingly appreciated that NSCLC is a phenotypically and genomically diverse malignancy. The exploitation of NGS has allowed a better understanding of tumor heterogeneity and its resulting evolution. The Charles Swanton lab spearheaded this work through their United Kingdom observational longitudinal registry called TRACERx (Tracking Cancer Evolution through Therapy)–Lung (NCT01888601) [6,7]. The initial report from this group included 100 patients who underwent upfront surgical resection for lung cancer (pathologic stage IA [*n* = 26], IB [*n* = 36], II [*n* = 24], and III [*n* = 14]); 61% of patients had LUAD, and only 12% were never-smokers. Following multiregional tumor sampling, whole-exome sequencing was performed. Analysis of the TRACERx-Lung registry revealed robust tumor heterogeneity, as demonstrated by substantial intratumoral subclonal somatic mutations and somatic copy number alterations (SCNAs). Importantly, if multiregional sampling had not been performed, 76% of subclonal mutations would have been incorrectly classified as clonal. Tumors with a high proportion of subclonal mutations had a higher associated risk of recurrence or death after complete resection. Causes of intratumoral heterogeneity include specific mutational processes (smoking and APOBEC signatures), increasing chromosomal instability (CIN), and whole-genome doubling (WGD), which is primarily clonal. Another important aspect of understanding the etiology of tumor heterogeneity is the discernment of clonal and subclonal driver alterations and when they occur. Many gene alterations occur early and are clonal (e.g., *EGFR*, *KRAS*, *MET*, *BRAF*, and *TP53*). In the TRACERx-Lung registry, alterations in specific driver genes were primarily clonal and almost always occurred before genome duplication, suggesting a role in tumor initiation and early evolution [6]. Other specific genomic alterations are later events, are subclonal, and are more frequently enriched in associated metastases, which emphasizes the importance of ongoing CIN in these tumors. While 20% of tumors had subclonal targetable alterations in the TRACERx study, 71% also possessed a clonal mutation, indicating the importance of clonal mutations in selecting targeted therapies [6].

Further analysis of the TRACERx-Lung cohort has shown that SCNA, but not mutation, predicts poor outcomes after surgical resection. Our group has reported similar observations—namely, that increased fraction of genome altered (FGA; the percentage of the genome exhibiting copy number gains or losses) is strongly associated with recurrence after R0 resection in patients with LUAD (*N* = 426). We also observed that *TP53* and *SMARCA4* mutations were independently associated with recurrence, even when controlling for pathologic stage and high-risk pathologic features [8].

The TRACERx-Lung study and similar studies were the first to use the power of NGS to better understand tumor biology, inform prognostication and prediction of recurrence, and identify therapeutic vulnerabilities and mechanisms of therapeutic resistance. As more studies use NGS approaches to explore tumor heterogeneity and evolution, there will be an increasing number of opportunities to clinically leverage this growing body of knowledge.

## 3. Understanding the Biology of LUAD Histologic Subtypes

On the basis of the recommendations from a multidisciplinary panel, a working group of the International Association for the Study of Lung Cancer, American Thoracic Society, and European Respiratory Society jointly proposed a classification system in 2011 to include predominant histologic subtyping of invasive LUAD [9]. Since then, numerous studies have demonstrated an association between histologic subtype and patient prognosis, with micropapillary (MIP) and solid (SOL) histologic subtypes associated with more recurrences and worse outcomes [10,11,12,13]. In parallel to the LUAD histologic subtype classifications, NGS has increasingly been used to elucidate tumor biologies and inform prognosis. Despite these known associations between both LUAD predominant histologic subtypes and genomic features derived from NGS with prognosis, few data exist regarding the relationship between genomic alterations for each subtype and their impact on clinical outcomes.

**Figure 1:**
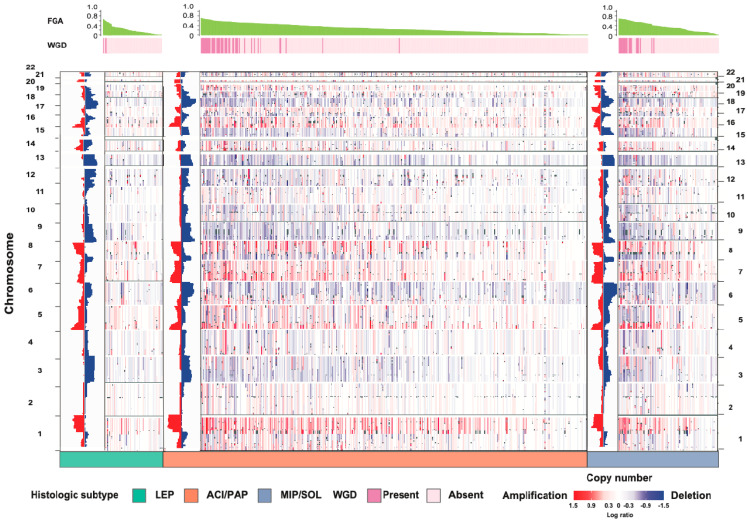
Analysis of copy number alterations by histologic subtype of invasive lung adenocarcinoma. The copy number heat map with amplifications (red) and deletions (blue) by histologic subtype, arranged by decreasing FGA. ACI, acinar; FGA, fraction of genome altered; LEP, lepidic; MIP, micropapillary; PAP, papillary; SOL, solid; WGD, whole-genome doubling. (From *Journal of Thoracic Oncology*, Caso, R.; Sanchez-Vega, F.; Tan, K.S.; Mastrogiacomo, B.; Zhou, J.; Jones, G.D.; Nguyen, B.; Schultz, N.; Connolly, J.G.; Brandt, W.S., “The underlying tumor genomics of predominant histologic subtypes in lung adenocarcinoma.” Vol. 15, 1844–1856. Copyright ^©^ (2020) Elsevier. Reprinted with permission).

We recently performed broad-panel NGS on 604 surgically resected nonmucinous LUAD tumors to investigate multiple tumor genomic features and their association with predominant histologic subtype. Features including oncogenic pathway alterations, CIN, mutational signatures, and targetable driver gene alterations were examined in relation to both subtype and tumor recurrence. We found individual gene differences between subtypes, including more alterations in *EGFR*, *RBM10*, and *TERT* in lepidic (LEP) tumors, compared with acinar (ACI) and papillary (PAP) tumors and MIP and SOL tumors, and more alterations in *TP53*, *SETD2*, *MGA*, and *SMARCA4* in MIP and SOL tumors, compared with ACI and PAP tumors and LEP tumors [14]. Other studies have found that adenocarcinoma in situ and minimally invasive adenocarcinoma have fewer mutations and oncogenic drivers than invasive tumors [15]. Of the known actionable oncogenic driver mutations, the frequency of *EGFR* alterations was highest in LEP tumors and the frequency of *BRAF* alterations was highest in MIP and SOL tumors. Interestingly, no statistically significant differences in *KRAS* G12C mutation, a known factor of poor prognosis, were observed among subtypes [14]. However, *KRAS* mutations were found to be more frequent in mucinous LUADs than in nonmucinous LUADs [16]. Within LUAD subtypes, both tumor mutation burden (TMB) and FGA were found to increase with subtype invasiveness. Importantly, we found that multiple measures of CIN, including copy number amplification, FGA, and WGD, were statistically significantly higher with more-aggressive histologic subtypes (Figure 1) [14]. Examination of the TRACERx cohort also demonstrated that higher CIN was associated with higher metastatic risk and shorter disease-free survival (DFS) [6].

**Figure 2:**
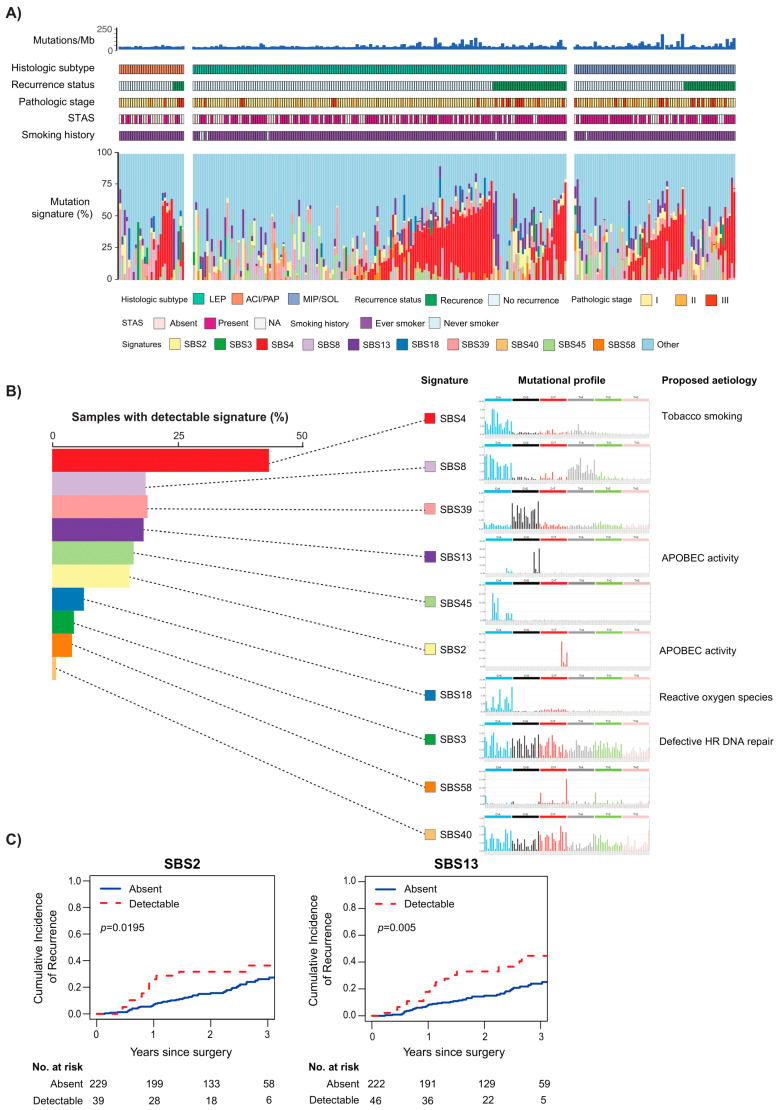
Analysis of somatic mutational signatures. (**A**) Bar plots of detectable mutational signatures (colored) by individual tumors. Tracks above the bar charts indicate (1) somatic mutations, (2) histologic subtype, (3) recurrence status, (4) pathologic stage, (5) tumor STAS, and (6) smoking history. (**B**) Distribution of select detectable signatures across all tumors and their representative mutational profiles and the proposed cause. (**C**) Cumulative incidence of postresection recurrence curves according to SBS2 and SBS13 mutational signature status across all patients. ACI, acinar; HR, homologous recombination; LEP, lepidic; MIP, micropapillary; NA, not available; PAP, papillary; SBS, single-base substitution; SOL, solid; STAS, spread through air spaces. (From *Journal of Thoracic Oncology*, Caso, R.; Sanchez-Vega, F.; Tan, K.S.; Mastrogiacomo, B.; Zhou, J.; Jones, G.D.; Nguyen, B.; Schultz, N.; Connolly, J.G.; Brandt, W.S., “The underlying tumor genomics of predominant histologic subtypes in lung adenocarcinoma.” Vol. 15, 1844–1856. Copyright ^©^ (2020) Elsevier. Reprinted with permission).

In addition to chromosomal alterations and changes at the individual gene level, clinical phenotypes are driven by alterations in known oncogenic pathways [17]. We observed that, among surgically resected LUAD tumors, RTK/RAS pathway alterations were associated with LEP-predominant tumors, and MIP- and SOL-predominant tumors had more alterations in the p53, Wnt, and Myc pathways, compared with ACI and PAP tumors and LEP tumors. Similarly, in concordance with previously described findings regarding TMB, an increase in the number of altered pathways was also associated with increased invasiveness and worse DFS across stages [14,18]. Within individual subtypes, cell cycle and PI3K pathway alterations were associated with poor prognosis in patients with ACI and PAP tumors, and PI3K alterations were associated with a higher incidence of recurrence in patients with MIP and SOL tumors. We next investigated somatic mutational signatures by histologic subtype (Figure 2A). Median somatic mutations increased with more-aggressive histologic subtype. We also evaluated detectable somatic mutational signatures across all tumors (Figure 2B). This investigation of mutational signatures associated with postresection recurrence revealed that SBS2 and SBS13 were associated with an increased risk of tumor recurrence (Figure 2C). SBS2 and SBS13 are both signatures associated with the APOBEC family of cytidine deaminases (APOBEC3A and APOBEC3B). These enzymes are known to fuel tumor diversity, subclonal evolution, and therapeutic resistance and were found with increasing frequency in more-invasive subtypes [19]. Interestingly, the combination of high TMB and the presence of APOBEC mutational signatures was reported to predict immunotherapy response in patients with NSCLC [20]. When investigating the relationship between known LUAD targetable alterations with existing therapies and histologic subtype, we found an inverse relationship between the frequency of targetable alterations and subtype invasiveness. MIP and SOL tumors had the lowest frequency of targetable LUAD alterations (27%), compared with ACI and PAP tumors (36%) and LEP tumors (41%).

While many questions remain with respect to the use of predominant histologic subtypes in LUAD tumors, NGS has provided several clues to help understand the differences in tumor biology and associated clinical outcomes. In general, when stratifying on the basis of subtype invasiveness, more-invasive LUAD subtypes (MIP, SOL) have higher TMB, more CIN, and altered oncogenic pathways. In addition, specific targetable genomic alterations vary in frequency across subtypes, with targetable *EGFR* mutations more common in LEP tumors than in ACI and PAP or MIP and SOL tumors and fewer level I actionable mutations in MIP and SOL tumors than in ACI and PAP or LEP tumors. Collectively, these findings provide important information to better understand the underpinnings of varying clinical phenotypes observed across the spectrum of LUAD histologic subtypes.

## 4. NGS for Prognosis and Prediction of Recurrence after Surgery

Although NGS now plays an essential role in identifying actionable genomic alterations and guiding subsequent therapies in advanced-stage NSCLC, its role in early-stage disease is less well defined. In patients with completely resected early-stage disease, recurrence remains the main determinant of long-term survival, with 5-year overall survival (OS) from 73% to 90% for stage I and 41% to 65% for stage II–IIIA disease [21]. At present, decisions to administer induction or adjuvant therapy are based solely on tumor-node-metastasis (TNM) staging and are agnostic to tumor genomic information. However, there is a limited survival benefit of 5% at 5 years for both adjuvant and neoadjuvant chemotherapy [22,23,24]. Therefore, improving the ability to predict the likelihood of recurrence and identify which patients could potentially benefit from adjuvant or neoadjuvant therapy is of crucial importance in improving the prognosis of patients with resectable early-stage NSCLC.

**Figure 3:**
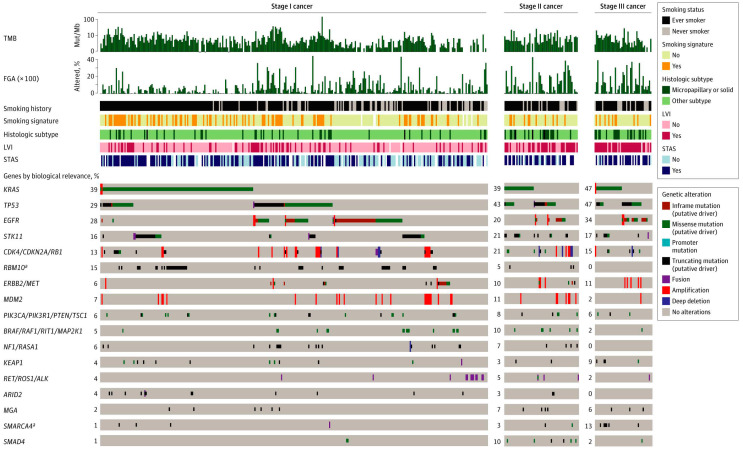
Oncoprint by pathologic stage with annotated clinicopathologic variables. Genes are grouped by biological relevance in lung adenocarcinoma. FGA, fraction of genome altered; LVI, lymphovascular invasion; Mut/Mb, mutations per megabase; STAS, spread through air spaces; TMB, tumor mutation burden. ^a^ *p* < 0.05, false-discovery rate, for difference in alteration frequency between stages using Fisher’s exact test. (From *JAMA Surgery*, Jones, G.D.; Brandt, W.S.; Shen, R.; Sanchez-Vega, F.; Tan, K.S.; Martin, A.; Zhou, J.; Berger, M.; Solit, D.B.; Schultz, N., “A genomic-pathologic annotated risk model to predict recurrence in early-stage lung adenocarcinoma.” Vol. 156, e205601–e205601. Copyright ^©^ (2021) American Medical Association. Reprinted with permission).

**Figure 4:**
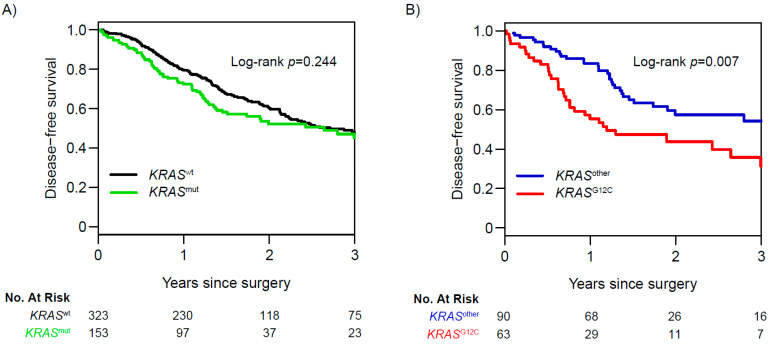
Association between *KRAS* mutation status and disease-free survival (DFS) in The Cancer Genome Atlas cohort (*N* = 476). (**A**) Three-year DFS for all *KRAS* mutant (*KRAS*^mut^) tumors versus *KRAS* wild-type (*KRAS*^wt^) tumors. (**B**) Three-year DFS for *KRAS*^G12C^ tumors versus all other *KRAS* mutant tumors (*KRAS*^other^). (From *Clinical Cancer Research*, Jones, G.D.; Caso, R.; Tan, K.S.; Mastrogiacomo, B.; Sanchez-Vega, F.; Liu, Y.; Connolly, J.G.; Murciano-Goroff, Y.R.; Bott, M.J.; Adusumilli, P.S., “KRASG12C mutation is associated with increased risk of recurrence in surgically resected lung adenocarcinoma.” Vol. 27, 2604–2612 Copyright ^©^ (2021) American Association for Cancer Research. Reprinted with permission).

NSCLC is often driven by specific oncogenic alterations, and while they represent potential therapeutic targets, they also provide important information regarding tumor biology and the likelihood of recurrence (Figure 3). At the individual gene level, alterations in genes such as *TP53*, *SMARCA4*, *CDKN2A*, *CTNNB1*, *ALK*, *ROS1*, and *RET*, among others, have been associated with higher levels of recurrence in patients with stage I–III disease [8,25,26,27]. In contrast, alterations in *EGFR* and the RNA-binding protein gene *RBM10* have been associated with a better prognosis [8,25]. As an example of how individual gene alterations may be used, we recently examined the prognostic value of *KRAS* G12C mutations. *KRAS* mutations are among the most common alterations in NSCLC, and the *KRAS* G12C mutation, in particular, has specific importance due to its association with poor outcomes and the recent development of specific G12C inhibitors. We reported that, in 604 patients with LUAD who underwent complete surgical resection, *KRAS* G12C mutation was an independent predictor of worse 3-year DFS, compared with wild-type and other *KRAS* mutations. We then validated our observations using The Cancer Genome Atlas cohort (*N* = 426) (Figure 4A,B) [28]. Although it was long thought that such *KRAS* mutations were undruggable, recent drug discovery efforts have resulted in Federal Drug Administration approval of sotorasib (AMG-510), a specific *KRAS* G12C inhibitor for patients with locoregionally advanced metastatic NSCLC.

Although specific gene alterations can provide prognostic information, examination of the tumor mutational status at the chromosome and genome levels offers additional clues to the biology of a given tumor. TMB is a potential, albeit controversial, biomarker for resected NSCLC; some studies have demonstrated favorable outcomes with high TMB, and, conversely, others have shown worse DFS and OS with high TMB [29,30]. Although its role as a prognosticator is a matter of debate, TMB has been shown to predict response to immunotherapy for multiple solid tumors, including NSCLC [31,32]. Perhaps more importantly, high TMB has been associated with other features associated with poor prognosis, including smoking history, invasive subtype, spread through air spaces, and node positivity. In addition, aggressive mutation profiles, such as *KRAS* G12C mutants, have an overall higher TMB than less-aggressive tumors [8,28]. Changes at the chromosomal level have also provided insight into the development and progression of different cancers. Alteration to the tumor copy number has been shown to be a prognostic factor across multiple solid tumors, with increasing rates of alteration associated with recurrence and death [33]. Copy number alterations (CNAs) and, in particular, genome doubling are early events in lung cancer evolution, followed by extensive subclonal diversification. As a result, somatic copy number amplifications have been linked to the development of metastases and worse DFS, while other markers of CIN, including FGA and WGD, have been linked to poor prognosis and more-aggressive clinicopathologic features [6,8].

Attempts to identify prognostic signatures associated with tumor recurrence have primarily focused on gene expression studies and have mostly used microarray expression profiling [34,35]. However, these approaches fail to address transcriptomic heterogeneity, which is related to tumor sampling bias. To address this issue and to better refine biomarker discovery, Biswas and colleagues from the TRACERx group used an RNA-seq approach to ultimately generate a 23-gene prognostic signature called ORACLE [34]. The authors found that early-event clonal DNA copy number amplifications primarily drive gene alterations expressed homogeneously in the tumor and that ORACLE was prognostic in pathologic stage I LUAD.

**Figure 5:**
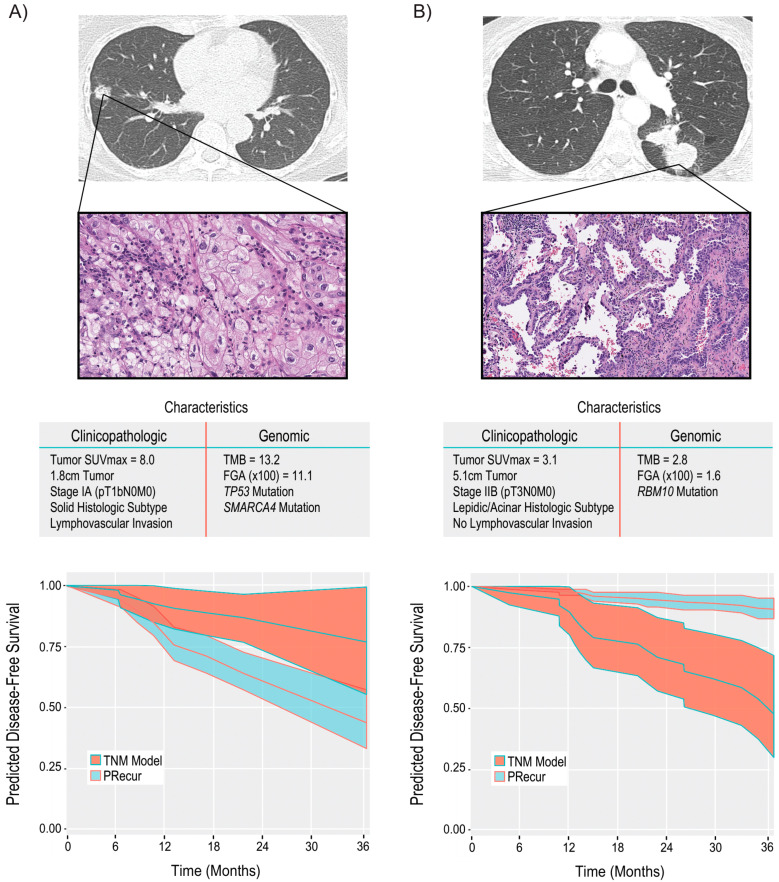
Computational machine-learning prediction model (PRecur) for disease-free survival applied to two patient scenarios. (**A**) Patient with a small, 1.8-cm tumor (pT1bN0M0, stage IA). Three-year disease-free survival curves were predicted by PRecur versus the tumor-node-metastasis model for all patients with pT1bN0M0 disease in our cohort (*n* = 136). (**B**) Patient with a large, 5.1-cm tumor (pT3N0M0, stage IIB). Three-year disease-free survival curves were predicted by PRecur versus the tumor-node-metastasis model for all patients with pT3N0M0 disease in our cohort (*n* = 53). FGA, fraction of genome altered; TMB, tumor mutation burden. (From *JAMA Surgery*, Jones, G.D.; Brandt, W.S.; Shen, R.; Sanchez-Vega, F.; Tan, K.S.; Martin, A.; Zhou, J.; Berger, M.; Solit, D.B.; Schultz, N., “A genomic-pathologic annotated risk model to predict recurrence in early-stage lung adenocarcinoma.” Vol. 156, e205601–e205601. Copyright ^©^ (2021) American Medical Association. Reprinted with permission).

To date, single-gene genomic alterations and RNA signature-based approaches have demonstrated modest prognostic value but have not been able to predict clinical outcomes, including tumor recurrence, following surgery. Given the limitations of the TNM staging system to predict tumor recurrence after complete (R0) resection, we recently examined a combination of tumor genomic and clinicopathologic variables in patients with LUAD, of whom 75% had pathologic stage I disease [8]. We used broad-panel NGS (MSK-IMPACT) to sequence 426 completely resected LUAD tumors, with the primary endpoint of recurrence-free survival. We observed that FGA, but not TMB, was associated with recurrence. To develop our prediction model (Predict Recurrence, or PRecur), we integrated high-risk clinicopathologic features and tumor genomics using a publicly available machine learning algorithm. We found that differentiation into low-, moderate-, and high-risk groups for development of recurrence by PRecur outperformed TNM classification for resectable early-stage LUAD (Figure 5). We then externally validated our findings using The Cancer Genome Atlas database. In renal and breast cancers, the use of broad-panel NGS alongside machine learning algorithms has already resulted in the creation of genomically annotated risk-stratification models [35,36]. The development of contemporary risk-stratification models for patients is essential in order to increase the appropriate use of adjuvant therapies and, conversely, avoid the use of inappropriate therapies. In addition, it is plausible that, in the postoperative setting, risk models based on both genomic and pathologic features, such as PRecur, combined with circulating tumor DNA (ctDNA) analysis may offer the best risk assessment for postoperative recurrence [8].

## 5. Plasma ctDNA for Early Detection of Lung Cancer and Monitoring for MRD

Owing to technological advances, NGS has expanded from the molecular characterization of lung cancers using tumor tissue to include the use of liquid biopsies for plasma ctDNA. At present, the National Comprehensive Cancer Network guidelines recommend ctDNA assays to test for driver mutations, such as *EGFR*, *ALK*, *ROS1*, *KRAS*, *BRAF*, *MET*, *RET*, and *NTRK*, in instances where a patient is unfit to undergo invasive tumor biopsy, as well as for testing for acquired resistance mutations, such as *EGFR* T790M. While this mostly applies to advanced NSCLC, the potential to detect MRD in patients with early-stage disease represents a paradigm-changing use for this technology.

In comparison to advanced-staged disease, there are multiple limitations to the use of ctDNA in early stages. However, key to any use of these assays is the ability to detect ctDNA at very low concentrations after complete surgical resection. As a result, multiple studies have looked to identify factors associated with ctDNA detection. In a TRACERx study of early-stage NSCLC, Abbosh et al. examined clinicopathologic determinants of ctDNA detection in 100 patients and found that, in preoperative samples, at least 2 single-nucleotide variants (SNVs) were detected in ctDNA in 46 of 96 cases (48%) and 1 SNV was detected in 12 additional cases. Among these samples, detection of ctDNA was associated with histologic subtype—ctDNA was identified in 97% of lung squamous cell carcinomas versus 19% of LUADs. Other factors associated with detection of ctDNA included tumor necrosis, lymph node involvement, lymphovascular invasion, increased pathologic tumor size, Ki67 indices, and total cell-free DNA (cfDNA) [37]. In another retrospective study of 40 patients with localized NSCLC, ctDNA was detected preoperatively in 93% of patients, and ctDNA was associated with metabolic tumor avidity on FDG-PET [38]. Genomic factors, including clonality and cancer cell fraction, were also associated with detection of ctDNA [37,39]. Among detectable mutations in the TRACERx cohort, clonal SNVs were more often detected than subclonal SNVs, and, in concordance with this finding, clonal SNVs had a higher variant allele fraction than their subclonal counterparts [37]. However, although driver mutations are largely truncal and clonal in tumor evolution, among LUADs in the TRACERx study, mutations in *KRAS*, *EGFR*, and *TP53* were not associated with improved detection of ctDNA. Using a CAPP-Seq assay, Chaudhuri et al. detected both driver and nondriver mutations in ctDNA samples, with the most frequently detected mutations found in surveillance samples (mutations in *TP53*, *KRAS*, *EGFR*, and *KEAP1*). However, the authors also observed that many nondriver genes were clonal, which highlights the importance of these genes in the potential detection of ctDNA [38].

Detecting MRD is of great interest because, despite complete surgical resection of early-stage NSCLC, 30% to 70% of patients will develop recurrence and die secondary to disease progression. In the LACE pooled analysis, a 5-year absolute survival benefit of only 5.4% was seen with cisplatin-based chemotherapy after complete resection of NSCLC, suggesting that the majority of patients who receive adjuvant chemotherapy are exposed to its toxicities without substantial benefit [23]. While TNM staging remains the main predictor of benefit from adjuvant therapy, personalized biomarkers for risk stratification of recurrence are urgently needed to help distinguish patients who may benefit from adjuvant therapy from those who may not. By the use of personalized tumor-informed assays, ctDNA liquid biopsies have been shown to successfully identify MRD in patients with breast and colon cancers, with good specificity [35,40]. In NSCLC, plasma ctDNA surveillance has been investigated as a potential means of detecting MRD not identifiable by standard imaging; however, this is dependent on the ability to detect very low levels of ctDNA present following resection. More recently, novel NGS bioinformatics and proteomics approaches to detect ctDNA in patients with early-stage lung cancer have shown potential as tools for monitoring for MRD after surgical resection, as well as screening tools for early cancer detection [36,38,41,42,43].

In patients with disease recurrence, detection of ctDNA often predates radiographic evidence of recurrence, providing an opportunity for earlier escalation of treatment, when systemic therapy may be more effective. In patients with negative or equivocal follow-up CT scans, detection of MRD by ctDNA was found to reliably predict recurrence [38]. However, after surgical resection, on average, fewer mutations and mutations at lower variant allele fractions were identified in ctDNA, compared with before treatment, highlighting the need to track multiple mutations per patient using tumor-informed bespoke assays to increase the sensitivity of detection [38,39]. Postresection detection of ctDNA provides the potential for both early identification of recurrence and improved risk stratification. Quantification of postresection changes in ctDNA levels has been shown to discriminate high- and low-risk patients by identifying indolent versus aggressive MRD [44]. Additionally, detection of ctDNA alone has been shown to be associated with risk of recurrence, disease-specific survival, and OS in patients with resected NSCLC [37,38].

Future uses of ctDNA assays, in addition to monitoring for MRD, include guiding systemic therapy as cancer interception and improving screening methods in the absence of tumor genomic information. While plasma ctDNA is currently used to identify *EGFR* and other activating mutations to guide precision therapy in the metastatic disease setting, clonal hematopoiesis is a pervasive source of nontumor-derived cfDNA and remains a major challenge in the clinical interpretation and utility of ctDNA assays [45,46,47,48]. In the setting of MRD, ctDNA sequencing has been shown to potentially identify resistance to adjuvant therapy and markers of response to immune therapy. TMB measured from plasma has been proposed as a potential biomarker of response to immune checkpoint inhibitor (ICI) treatment, with high TMB identifying patients who have clinically significant improvements in outcomes with ICI treatment [49]. In addition, the pretreatment ctDNA level is associated with response to ICI treatment and, when used as a surveillance tool, can predict progression in patients with response to programmed cell death protein 1 or programmed cell death ligand 1 blockade [50,51]. The use of machine learning algorithms, combined with improved NGS methods, can identify ctDNA at very low levels in early-stage cancers, at rates similar to those for tumor-informed detection methods, but in the absence of tumor sequencing information [43]. These algorithms, which use sequencing fragment information, mutational signatures, and matched white blood cell filtering to help identify ctDNA from nontumor variants resulting from clonal hematopoiesis and the germline, can distinguish patients with early cancers from healthy controls [43]. Novel assays using a variety of methods, including combining the discriminative value of proteomics, fragmentomics, methylation, and nucleosomes with ultrasensitive ctDNA analysis to improve early detection of cancer, are being developed [36,41,42,52,53]. At present, many hurdles to using ctDNA assays more broadly in the management of NSCLC exist. These include technological shortcomings that limit the ability to detect rare variants and biological processes, including clonal hematopoiesis and germline alterations that contribute to the cfDNA pool. In fact, the majority of all cfDNA mutations have been found to be consistent with clonal hematopoiesis, demonstrating the critical need to filter variants in order to correctly interpret cfDNA analyses [48]. However, the recent advances in NGS technology underline the potential for early detection of lung cancer, monitoring of MRD to detect and intercept recurrence early, and the development of interventions that target specific alterations and improve survival (Table 1).

## 6. Differentiation between Separate Primary Lung Cancers (SPLCs) and IPMs

The ability to discern whether a lung cancer is an SPLC or an IPM has become increasingly important given the increase in multifocal LUAD. The criteria proposed by Martini and Melamed in 1975 have been the most widely adopted, although molecular pathology assays have increasingly been used to supplement the older histopathologic criteria [54,55]. Early molecular methods included microsatellite and loss-of-heterozygosity analysis, comparative genomic hybridization arrays, and *TP53* gene mutation status. More recently, oligogene panels for hotspot mutations in 2 to 5 major driver genes, as well as limited-panel (i.e., 50-gene) NGS, have been performed [56]. Unfortunately, given the small number of genes being examined, these approaches are limited in scope and granularity; therefore, they cannot fully address the clinical question of SPLC or IPM [56,57]. In their study examining the use of a combined histologic and molecular approach to distinguish SPLCs from IPMs, Mansuet-Lupo et al. found that only 9% of all patients were inconclusive based on their NGS panel, as opposed to 28% of patients who would have been inconclusive using a 5-gene approach [58]. We leveraged our 468-gene broad-panel NGS platform and found a marked improvement in our ability to discern NSCLC clonality in multiple lung cancers in the same patient.

We performed prospective histologic comparison and genomic profiling in 76 pairs of tumors to predict whether the pairing represented IPMs or SPLCs. Using prospective histologic comparison, we predicted that 20 tumor pairs (26%) were IPMs on the basis of similar morphologic appearance and 56 tumor pairs (74%) were morphologically different and were therefore SPLCs. When looking at only adenocarcinoma pairs (*n* = 70), 19 were predicted to represent IPMs, and 51 were predicted to represent SPLCs. In these 76 patients, genomic profiling of 128 tumors by MSK-IMPACT yielded a median of 8 somatic alterations per tumor (range, 1–47), and a major oncogenic driver alteration (e.g., *EGFR*, *KRAS*, *ALK*, *ROS1*, or *MET* exon 14) was identified in 107 tumors (84%). According to NGS, 25 tumor pairs were classified as IPMs (24 definite and 1 high probability), and 51 were definite SPLCs. When comparing the prospective histologic prediction and final molecular classification, 17 of 76 tumor pairs (22%) had discordant results. The discordance rate was higher for IPMs (11/25 [44%]) than for SPLCs (6/51 [12%]) (*p* < 0.001). In another study examining the use of NGS to differentiate SPLCs from IPMs, the authors found histologic review alone misclassified 27% of the 33 evaluated tumor pairs [59].

**Figure 6:**
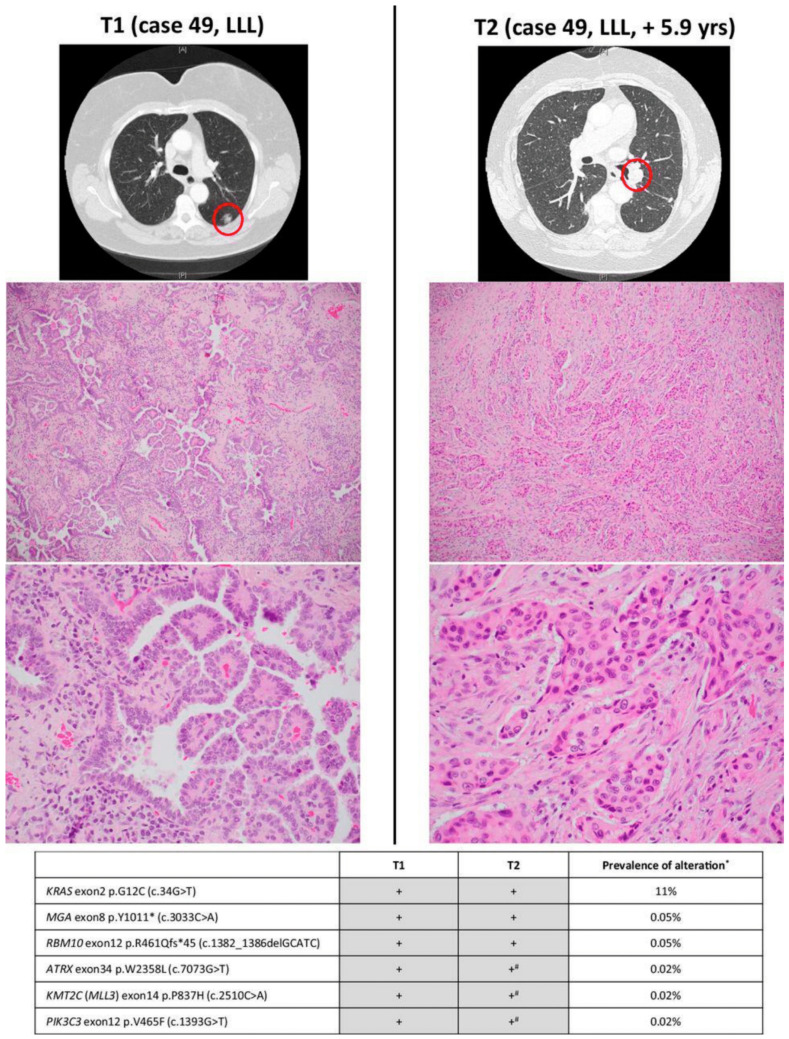
Radiologic and pathologic appearances and next-generation sequencing profile of an intrapulmonary metastasis, showing histologic progression consisting of entirely solid pattern. #, mutations present (subthreshold) in T2 on manual review *; prevalence is based on MSK-IMPACT results for 4119 non-small cell lung cancers in the cBioPortal database. On the basis of combined prevalence of each mutation, the odds of coincidental co-occurrence are 1.52 × 10^−37^. LLL, lower left lobe. (From *Clinical Cancer Research*, Chang, J.G.C.; Alex, D.; Bott, M.; Tan, K.S.; Seshan, V.; Golden, A.; Sauter, J.L.; Buonocore, D.R.J.; Vanderbilt, C.M.; Gupta, S., “Comprehensive NGS unambiguously distinguishes separate primary lung carcinomas from intra-pulmonary metastases: Comparison with standard histopathologic approach.” Vol. 25(23), 7113–7125. Copyright ^©^ (2019) American Association for Cancer Research. Reprinted with permission).

Interestingly, we observed that the use of identical *KRAS* mutations to determine clonality was often incorrect. In our study, we found that the use of broad-panel NGS helped to discriminate unrelated tumors (SPLCs) that shared a single common hotspot mutation by chance. In specific populations, this can be especially problematic [58]. For example, in smokers with tumors sharing a *KRAS* G12C mutation or in never-smokers with tumors sharing an *EGFR* exon 21 L858R mutation, the odds of co-occurrence by chance can be as high as 1 in 17. In our series, shared *KRAS* mutations were almost as likely to occur coincidentally in SPLCs as in IPMs [55]. As an example, a patient underwent a sublobar resection of a peripheral left lower lobe LUAD and then, 5.9 years later, developed a new left lower lobe LUAD that had increased SOL and MIP histologic subtyping. Although this was thought by the clinicians to be an SPLC, NGS confirmed that it was an IPM (Figure 6). Other groups have recently reported similar findings using CNA [60].

While histologic assessment is accurate in the majority of cases, these findings demonstrate the limitations, with discordance in approximately one-fifth of cases. Limitations of large-panel NGS platforms include availability, costs, and turnaround time. Because of the associated costs and the significant bioinformatic, computational, and personnel resources required to analyze and interpret the data, not all institutions currently offer NGS testing [55]. As a result, others have proposed smaller gene panels that can identify commonly mutated genes and treatment algorithms based on both molecular and genomic analysis [56,57,58,61]. Notwithstanding these limitations, broad-panel NGS has become the gold-standard for determining whether multiple lung cancers are SPLCs or IPMs. The exact number of cancer-related genes that need to be sequenced remains an open question, but as noted in other studies, smaller gene panels of 50 and 182 genes were noninformative in 28% and 14% of cases, respectively [59]. Moving forward, a comprehensive diagnostic approach that incorporates both histologic subtype and NGS will be essential to discriminate multiple NSCLCs by providing robust confirmation of tumor clonality and identifying actionable mutations.

## 7. Use of NGS to Guide Induction and Adjuvant Therapies for Operable Lung Cancer

There are currently at least 10 targetable oncogenic drivers in LUAD; these include mutations in *EGFR*, *BRAF^V600E^*, *MET* exon 14, and *HER2*; rearrangements in *ALK*, *RET*, *NTRK*, and *ROS1*; and amplification of *MET* and *HER2* [4,5,62]. Moreover, recent phase I clinical trials in advanced-stage disease have shown promising results targeting *KRAS* G12C mutant LUAD, which occurs in 13% to 16% of patients with LUAD [28,63]. Collectively, the percentage of LUAD cases with a targetable genomic perturbation, including *KRAS* G12C, is now 50% to 60% and may be higher in Asia, where the incidence of *EGFR* mutations is approximately 2–3 times the incidence observed in North America and Europe (35–45% vs. 15–25%) [64]. Studies designed to assess the safety and efficacy of the integration of targeted therapies into therapeutic algorithms for early-stage lung cancer have begun, and their progress is accelerating (Table 2).

### 7.1. Adjuvant Clinical Trials

The phase III FLAURA trial compared osimertinib with gefitinib or erlotinib in untreated *EGFR* mutation–positive (Ex19del or Ex21L858R) advanced-stage NSCLC and found overall superiority of osimertinib, with longer progression-free survival and OS [65,66]. On the basis of these findings, the randomized phase III ADAURA trial examined adjuvant osimertinib with or without adjuvant chemotherapy [67]. This trial demonstrated that, after complete resection of pathologic stage IB–IIIA LUAD, adjuvant osimertinib was well tolerated and produced a statistically significant improvement in DFS, with an overall 83% reduction in disease recurrence or death. Although important secondary endpoints of OS await data maturation, this study was the first to show that integration of targeted therapies on the basis of specific tumor genomic alterations can improve outcomes in patients with surgically resected NSCLC [68].

Given the low frequencies of the above noted oncogenic drivers, there are few planned adjuvant targeted therapy clinical trials. However, there is activity in the adjuvant space for ALK translocations, with two randomized phase III trials ongoing [62]. The ALINA trial (NCT03456076) is comparing 24 months of adjuvant alectinib with the standard of care (4 cycles of chemotherapy) in patients with stage IB–IIIA resected ALK-rearranged NSCLC. Adjuvant radiation is not permitted, and patients with N2 stage IIIA disease are excluded. The primary endpoint is DFS. In the ALCHEMIST trial (NCT02194738), patients with stage IB–IIIA resected ALK-rearranged NSCLC are randomized to either crizotinib or observation after completion of standard therapy, including chemotherapy and radiotherapy, when indicated. The primary endpoint is OS, with DFS as a secondary endpoint. The findings of these trials will not be available for at least several years.

A challenge for all adjuvant therapy trials is the lack of biomarkers to ascertain which patient(s) will benefit from adjuvant therapy and, conversely, which patients are unlikely to benefit. It is increasingly appreciated that MRD, as detected by ctDNA levels, is strongly associated with persistent disease after therapy and portends a worse overall outcome [37,38,39]. Upcoming clinical trials of adjuvant therapy based on MRD detection include MERMAID I, which will evaluate the effect of adjuvant durvalumab plus chemotherapy versus chemotherapy alone on DFS in patients with completely resected stage II–III NSCLC and MRD. This randomized phase II trial uses a bespoke, positive-ctDNA assay to determine MRD; the primary outcome is DFS. A similar study, MERMAID II, will examine adjuvant durvalumab alone versus placebo in patients with stage II–III NSCLC and MRD who have undergone curative-intent therapy (complete resection ± neoadjuvant and/or adjuvant therapy), who have no evidence of disease recurrence (as defined by Response Evaluation Criteria in Solid Tumors version 1.1), and who become MRD-positive during a 96-week surveillance period. The primary endpoint of MERMAID II is DFS in patients with tumors with >1% expression of programmed cell death ligand 1 on tumor cells.

### 7.2. Neoadjuvant Clinical Trials

Although there are few neoadjuvant clinical trials focused on specific tumor genomic alterations, the promising results of some adjuvant trials have resulted in an increasing number of studies in the neoadjuvant space [67]. The NEOADAURA study is a randomized phase III study designed to determine the major pathologic response rate in patients with clinical stage II–III NSCLC with an *EGFR* exon 19 deletion or exon 21 L858R mutation after neoadjuvant (1) chemotherapy, (2) osimertinib alone, or (3) osimertinib plus chemotherapy. Accrual is expected to be completed in 2024.

LEADER (NCT04712877) is a feasibility study designed to exploit the known targetable genomic alterations in the neoadjuvant setting. Led by the Lung Cancer Mutation Consortium, the Lung Cancer Research Foundation, and the Thoracic Surgery Oncology Group, LEADER is a screening study with the goal of determining the feasibility of comprehensive molecular profiling to detect actionable oncogenic drivers in patients with suspected early-stage lung cancers who are scheduled to undergo biopsy to establish the diagnosis of lung cancer before definitive surgery. Once actionable drivers are detected, patients will be enrolled in target-directed, harmonized neoadjuvant therapy trials with genomically matched treatments; if no drivers are detected, patients will be enrolled in other appropriate trials.

## 8. Conclusions

A better understanding of lung cancer genomics affords new opportunities to customize and enhance treatment strategies for patients. Although tumor genomic–based therapies began in the setting of locoregionally advanced and metastatic disease, there has been an increasing appreciation of the potential use of these therapies in earlier-stage disease. With time, this trend should only continue, with technological advancements leading to better understanding of NSCLC and more-personalized treatment approaches. In the future, NGS will play a critical role in further elucidating the genomic features of tumor biology, identifying new treatment targets, and improving risk-stratification in early-stage disease. However, with increasing amounts of information, other challenges arise, such as how to combine increasing clinical, pathologic, and genomic data into an optimal treatment strategy for individual patients. Already, new technologies such as machine-learning models for disease recurrence and the use of artificial intelligence in radiomics are beginning to address these concerns. Despite potential future challenges, over the past decade, technological advancements in NGS approaches, reduced costs of sequencing, and newly discovered applications (such as ctDNA) have resulted in considerable progress in tumor biology research and, more importantly, in driving new cancer care treatment paradigms.

## Figures and Tables

**Table 1:** Novel MRD and early detection assays in development for lung cancer.

Detection Assay	Developer	Application
CAPP-Seq	Stanford	MRD
Avenio	Roche	MRD
TEC-Seq	Johns Hopkins	MRD
Lung-CLiP	Stanford	Early detection
Cancer SEEK	Thrive/Exact Sciences	Early detection
Galleri	GRAIL	Early detection
Delfi	Delfi Diagnostics	Early detection
Signatera	Natera	MRD
PCM	ArcherDX	MRD
RaDaR	Inivata	MRD
PhasED-Seq	Foresight Diagnostics	MRD

MRD, minimal residual disease.

**Table 2:** NGS-driven adjuvant and neoadjuvant trials in patients with early-stage NSCLC undergoing surgical resection.

Name/NCT#	Trial Type	Genomic Target	Phase	Primary Outcome	No. of Patients	Population/Trial Design	Completion Date
ALCHEMIST/NCT02201992	Adjuvant	*ALK*	III-R	OS	168	Pathologic stage IB–IIIA/crizotinib × 24 months vs. placebo	2022
ALCHEMIST/NCT02193282	Adjuvant	*EGFR*	III-R	OS	450	Pathologic stage IB–IIIA/erlotinib × 24 months vs. placebo	2021
ALINA/NCT03456076	Adjuvant	*ALK*	III-R	DFS	255	Pathologic stage IB–IIIA/alectinib × 24 months vs. chemotherapy	2026
MERMAID 1/NCT04385368	Adjuvant	ctDNA	III-R	DFS	332	Pathologic stage II or III/durvalumab + SOC chemotherapy vs SOC chemotherapy	2026
MERMAID 2/NCT04642469	Adjuvant	ctDNA	III-R	DFS	284	Pathologic stage II or III/durvalumab vs placebo	2027
NEOADAURA/NCT04351555	Neoadjuvant	*EGFR*	III-R	MPR	328	Clinical stage II or III/SOC chemotherapy vs osimertinib + SOC chemotherapy vs osimertinib alone	2024
LEADER/NCT04712877	Neoadjuvant	Multiple	II	Feasibility	1000	Clinical stage IB–IIIA/tumor DNA for NGS before surgery	2026

ctDNA, circulating tumor DNA; DFS, disease-free survival; MPR, major pathologic response; NGS, next-generation sequencing; NSCLC, non-small cell lung cancer; OS, overall survival; SOC, standard of care.
